# Fucoidan from *Laminaria japonica* Ameliorates Type 2 Diabetes Mellitus in Association with Modulation of Gut Microbiota and Metabolites in Streptozocin-Treated Mice

**DOI:** 10.3390/foods12010033

**Published:** 2022-12-22

**Authors:** Chenxi Zhang, Jinhui Jia, Panpan Zhang, Weiyun Zheng, Xiaoming Guo, Chunqing Ai, Shuang Song

**Affiliations:** 1 School of Food Science and Technology, National Engineering Research Center of Seafood, Dalian Polytechnic University, Dalian 116034, China; 2 Shenzhen Key Laboratory of Food Nutrition and Health, Institute for Advanced Study, Shenzhen University, Shenzhen 518060, China; 3 National & Local Joint Engineering Laboratory for Marine Bioactive Polysaccharide Development and Application, Dalian Polytechnic University, Dalian 116034, China

**Keywords:** fucoidan, *Laminaria japonica*, gut microbiota and metabolites

## Abstract

Chronic diseases have been a leading cause of death worldwide, and polysaccharide supplementation is an effective therapeutic strategy for chronic diseases without adverse effects. In this study, the beneficial effect of *Laminaria japonica* fucoidan (LJF) on type 2 diabetes mellitus (T2DM) was evaluated in streptozocin-treated mice. LJF ameliorated the symptoms of T2DM in a dose-dependent manner, involving reduction in weight loss, water intake, triglyceride, blood glucose, cholesterol and free fatty acids, and increases in high-density lipoprotein cholesterol, catalase, glucagon-like peptide-1, and superoxide dismutase. In addition, LJF regulated the balance between insulin resistance and insulin sensitivity, reduced islet necrosis and β-cell damage, and inhibited fat accumulation in T2DM mice. The protective effect of LJF on T2DM can be associated with modulation of the gut microbiota and metabolites, e.g., increases in *Lactobacillus* and *Allobaculum*. Untargeted and targeted metabolomics analysis showed that the microbiota metabolite profile was changed with LJF-induced microbiota alterations, mainly involving amino acids, glutathione, and glyoxylate and dicarboxylate metabolism pathways. This study indicates that LJF can be used as a prebiotic agent for the prevention and treatment of diabetes and microbiota-related diseases.

## 1. Introduction

With changes in living conditions and lifestyle, chronic diseases have been an enormous threat to public health, e.g., obesity and diabetes, which bring heavy economic burden to patients and create poor health-related quality of life [1]. Diabetes is one of the fastest growing chronic diseases, characterized by hyperglycemia and glucosuria due to defects in insulin secretion and action. Diabetes is not a single disease and is related to some long-term complications, including neuropathy, nephropathy, and angiopathy. Diabetes increases the risk of liver disease, cardiovascular disease, and other disorders, leading to increased mortality in people with diabetes [2,3]. According to the prediction, more than 693 million people will be affected by diabetes by 2045, emphasizing the urgency for the treatment and prevention of diabetes [4].

In the past two decades, many drugs have been approved for the treatment of diabetes, e.g., insulin and its analogues [5], which increase the number of treatment options available for individuals with diabetes. However, the application of currently available anti-diabetic drugs has limited efficacy, adverse effects (e.g., nausea and diarrhea caused by metformin), and unfavorable delivery manner, even though it significantly reduces the mortality and morbidity of diabetes and associated diseases. In addition to seeking new drug molecules, a growing body of evidence indicates the efficacy and cost-effectiveness of nutrition therapy as an important component of diabetes care [6,7]. In 2010, the Academy of Nutrition and Dietetics published the nutritional recommendations for diabetes, and similar recommendations were published by the American Diabetes Association in 2013 [8]. It supports that functional food ingredients can be used in the management and therapy of diabetes and related diseases.

Polysaccharides, as high polymeric carbohydrate molecules, widely exist in animals, plants, and microorganisms, and have various bioactivities such as anti-obesity and anti-diabetes [9]. Clinical study showed that polysaccharides and oligosaccharides improved glucose–insulin metabolism and possessed promising hypoglycemic potential, similar to the current first-line drugs, without adverse effects [10]. *Laminaria japonica*, which is rich in polysaccharides, is one of the most popular marine foods in Asian countries [11]. *L. japonica* extract reduced fasting blood glucose (FBG), cholesterol, and triglyceride in diabetic patients without side effects, implying that some ingredients of *L. japonica* can benefit type 2 diabetes mellitus (T2DM) [12]. Recent studies demonstrated that polysaccharides from *L. japonica* improved experimentally induced T2DM in mice, e.g., reduction in weight loss, FBG, and triglyceride [13,14]. Current evidence supports the anti-diabetic effect of polysaccharides, but the exact mechanism needs more in-depth research.

In this study, the effect of *L. japonica* fucoidan (LJF) on T2DM was analyzed in streptozocin (STZ)-treated mice. LJF ameliorated the symptoms of T2DM in a dose-dependent manner, involving reduction in weight loss, cholesterol, FBG, triglyceride, and free fatty acids, and increases in glucagon-like peptide-1, high-density lipoprotein cholesterol, superoxide dismutase, and catalase. LJF improved the imbalance between insulin resistance and insulin sensitivity and reduced islet central necrosis and β-cells damage in mice. The beneficial effect of LJF can be associated with modulation of the gut microbiota, such as increases in *Lactobacillus* and *Allobaculum*. In addition, untargeted and targeted metabolomics analysis showed that the metabolites profile was changed with LJF-induced microbiota alterations, involving amino acids, glutathione, and glyoxylate and dicarboxylate metabolism pathways. It indicates that LJF can be developed as a prebiotic agent for the prevention and treatment of diabetes and other microbiota-related diseases.

## 2. Materials and Methods

### 2.1. Materials and Chemicals

*L. japonica* was purchased from Dalian Jiuyang Sea Products Co., Ltd. (Dalian, China). LJF was prepared according to the reported method [15], and the content of total sugar was ~68.8%, uronic acid was ~17.9%, and protein was ~3.7% (Table S1). LJF consisted of mannose, rhamnose, glucose, glucose, galactose, xylose, and fucose at the ratio of 4.0: 2.1: 1.0: 2.3: 7.5: 1.7: 20.4 (Figure S1). The molecular weight of LJF was ~89 kDa, and the content of the sulfate group was ~19.5% (Table S1). STZ, ammonium acetate, formic acid, and metformin (ME) were provided by Sigma (St. Louis, MO, USA). A bicinchoninic acid (BCA) protein assay kit was obtained from Solarbio (Beijing, China). Other reagents of analytical grade were obtained from Chemicals and Reagents Co., Ltd. (Beijing, China), and acetonitrile (HPLC grade) was purchased from Merck (Darmstadt, Germany).

### 2.2. Mice Experiment

#### 2.2.1. Ethics Statement

C57BL/6J male mice (6 weeks old, SPF, 20 ± 2 g) were provided by Liaoning Changsheng Biotechnology Co., Ltd. (Shenyang, China), and kept in the standard laboratory conditions (22 ± 2 °C, 50 ± 5% of relative humidity, and 12 h/12 h dark/light cycle). During the experiment, mice were fed with food and water ad libitum. This study was carried out in strict accordance with the Guidelines for the Care and Use of Laboratory Animals of the National Institutes of Health. The protocol was approved by the Laboratory Animal Ethics Committee of Dalian Polytechnic University (License No. DLPU2021047; Dalian, China). All efforts were made to minimize suffering.

#### 2.2.2. Experimental Protocol

**Figure 1:**
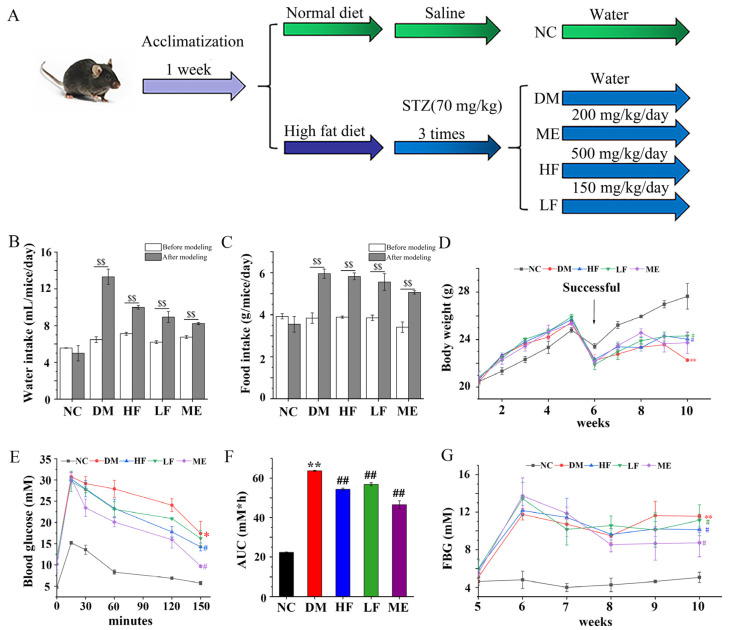
Effect of LJF on T2DM symptoms: process diagram of the mice experiment (**A**); intake of food (**B**) and water (**C**), ^$$^ *p* < 0.01, before and after modeling; body weight (**D**); blood glucose levels (**E**) and AUC (**F**) in the OGTT; FBG (**G**). * *p* < 0.05 and ** *p* < 0.01 vs. NC group, and ^#^ *p* < 0.05 and ^##^ *p* < 0.01 vs. DM group.

As shown in Figure 1A, mice were first divided into two groups: normal diet (NC, *n* = 8) and high fat/sugar diet (HFD, *n* = 32, 31% calories from fat). After five weeks, mice in the HFD group were intraperitoneally injected with STZ dissolved in citrate buffer at a dose of 70 mg/kg three times a week, and mice in the NC group were injected with citrate buffer as control. Three days after the third injection, mice in the HFD group with serum glucose > 11.1 mM were defined as T2DM mice. T2DM mice were subdivided into four groups (*n* = 8/group/two cages): (i) model group (DM), gavaged with normal water; (ii) ME treatment group (ME), gavaged with 200 mg/kg body mass of ME; (iii) high dosage of LJF group (HF), gavaged with 500 mg/kg body mass of LJF; and (iv) low dosage of LJF group (LF), gavaged with 150 mg/kg body mass of LJF. Body weight and FBG were monitored weekly. Liver weight was measured, and the ratio of liver weight/body weight was calculated.

### 2.3. Oral Glucose Tolerance Test (OGTT)

One week before the end of the experiment, mice were gavaged with 2 g/kg body mass of glucose [16]. Blood was harvested from the tail vein of mice at 0, 15, 30, 60, 120, and 150 min, and blood glucose levels were measured by a handheld glucose testing device (Roche, Germany). The area under the curve (AUC) was calculated.

### 2.4. Measurement of Biochemical Indexes in Serum

The assays of total cholesterol (TC), low- and high-density lipoprotein cholesterol (LDL/HDL-C), triglyceride (TG), glucagon-like peptide-1 (GLP-1), free fatty acids (FFA), aspartate aminotransferase (AST), alanine aminotransferase (ALT), and fasting serum insulin (FINS) were performed using commercial kits (Nanjing Jiancheng Biochemistry Institute, Nanjing, China). Homeostasis model assessment for insulin resistance (HOMA-IR) and insulin sensitivity index (ISI) were calculated.

### 2.5. Histology Analysis

Fresh pancreas tissues were fixed with 4% paraformaldehyde, dehydrated by gradient ethanol, cleared in xylene, and embedded with paraffin. Then, the tissue blocks were cut into 5 μm sections, stained with hematoxylin and eosin (HE), and checked by optical microscopy (Leica, Germany). In addition, the liver tissue blocks were cut into 9 μm sections and stained by Oil-Red O dye staining.

### 2.6. Analysis of Short Chain Fatty Acids (SCFAs)

SCFAs contents in mice feces were measured by gas chromatography as in our previous method [17].

### 2.7. Sequencing Analysis of the Gut Microbiota Composition

Sequencing analysis of the microbiota composition in mice feces was performed on an Illumina HiSeq platform according to our previous description [17]. Bacterial DNA was extracted, and the v3-v4 region of the 16S rRNA gene was amplified by PCR with universal primers (338F and 806R). After purification, the PCR products were used for the generation of sequencing library, and taxonomy assignment of OTUs was performed against Silva Database after processing raw reads. Bacterial richness and diversity were evaluated by Simpson, Chao1, and Shannon indexes, and the relation of microbiota communities between groups was analyzed using the unweighted pair–group method with arithmetic means (UPGMA) and principal coordinate analysis (PCoA). Linear discriminant analysis (LDA) was performed to identify key bacterial taxa that differed among groups (log10 > 3.5).

### 2.8. Untargeted and Targeted Metabonomics Analysis

Briefly, 100 mg of mice feces was dissolved in 500 μL of mixed solution consisting of acetonitrile/methanol/water (*v*/*v*/*v*, 2:2:1) and vortexed for 30 s. After centrifugation, the supernatant was collected for the follow-up experiment, and the residue was again reacted with the above solution to collect the supernatant. The internal standards were mixed with the collected supernatants and transferred to the sample vial for untargeted and targeted metabolomics analysis, as described in a previous study [18]. Data analysis was performed according to the reported method with slight modifications [19]. Mass Spectrometry-Data Independent Analysis software (http://prime.psc.riken.jp/; Accessed on 8 August 2022) was used for peak picking and alignment, and SIMCA14.1 was used for multivariate statistical analysis. Principal component analysis (PCA) and orthogonal partial least squares discriminant analysis (OPLS-DA) were performed for difference analysis between groups. MetaboAnalyst was used for metabolic pathway analysis, metabolite set enrichment analysis, and variable importance in the projection (VIP) value for the altered metabolites. Differential metabolites were identified based on fold-change (FC) threshold (FC > 2 and FC < 0.5) and VIP > 1.

### 2.9. Statistical Analysis

Data were expressed as mean ± standard deviation. Statistical analysis was performed using SPSS 20.0 software, and *p* < 0.05 was considered to be significant. The statistical significance was determined by one-way ANOVA, followed by post hoc Duncan’s multiple range test; * *p* < 0.05 and ** *p* < 0.01 vs. NC group, and ^#^ *p* < 0.05 and ^##^ *p* < 0.01 vs. DM group. Spearman analysis was performed to evaluate the relationship between key bacterial genera and physiological parameters or metabolites.

## 3. Results

### 3.1. LJF Regulated Weight Gain and Glucose Tolerance in Mice

Food and water intake was distinctly increased in the DM group after STZ injection, but body weight was reduced (Figure 1B–D). After treatment for 4 weeks, water intake was reduced in the LF/HF groups compared to the DM group, which was same as the ME group. There was no difference between the LF/HF and DM groups on food intake, but food intake was reduced in the ME group. Body weight was higher in the LF, HF, and ME groups compared to the DM group, and no difference was found between them. The OGTT result showed that blood glucose levels were higher in the DM group than in the NC group, which reached the highest level at 15 min (Figure 1E). Blood glucose declined rapidly to the initial state in normal mice, whereas it decreased slowly in the DM group. In contrast, blood glucose level decreased faster in mice fed with LJF or ME, and the effect of LJF on blood glucose was in a dose-dependent manner. The level of AUC was higher in the DM group than in the NC group, and LJF led to reduction in the AUC level in T2DM mice, which was higher than the ME group (Figure 1F). The level of FBG was higher in the DM group compared to the NC group, and LJF reduced FBG levels in T2DM mice in a dose-dependent manner (Figure 1G). These results indicated that LJF can be used as a prebiotic agent to ameliorate the classic symptoms of T2DM.

### 3.2. LJF Attenuated Lipid Metabolism Abnormality in T2DM Mice

**Figure 2:**
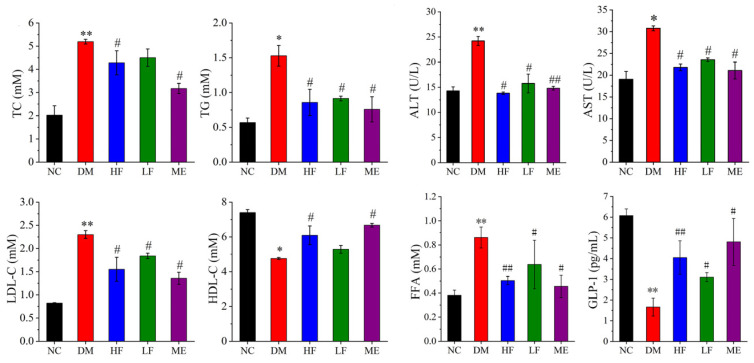
Effect of LJF on lipid-related indicators in T2DM mice, including TC, TG, ALT, AST, LDL-C, HDL-C, FFA, and GLP-1. * *p* < 0.05 and ** *p* < 0.01 vs. NC group, and ^#^ *p* < 0.05 and ^##^ *p* < 0.01 vs. DM group.

The contents of serum TG, LDL-C, TC, ALT, AST, and FFA were increased in the DM group than the NC group, and GLP-1 and HDL-C levels were reduced (Figure 2). In contrast, low and high dosages of LJF reduced the contents of serum LDL-C, TC, AST, TG, ALT, and FFA and increased HDL-C and GLP-1 levels in T2DM mice, which was same with the therapeutic effect of ME. It indicated that LJF effectively improved lipid metabolism abnormality in T2DM mice. Overall, high dosage of LJF appeared to have a slightly better effect on some parameters compared to low dosage, e.g., HDL-C and GLP-1.

### 3.3. LJF Improved Glycogen Content and Oxidative Stress in the Liver

**Figure 3:**
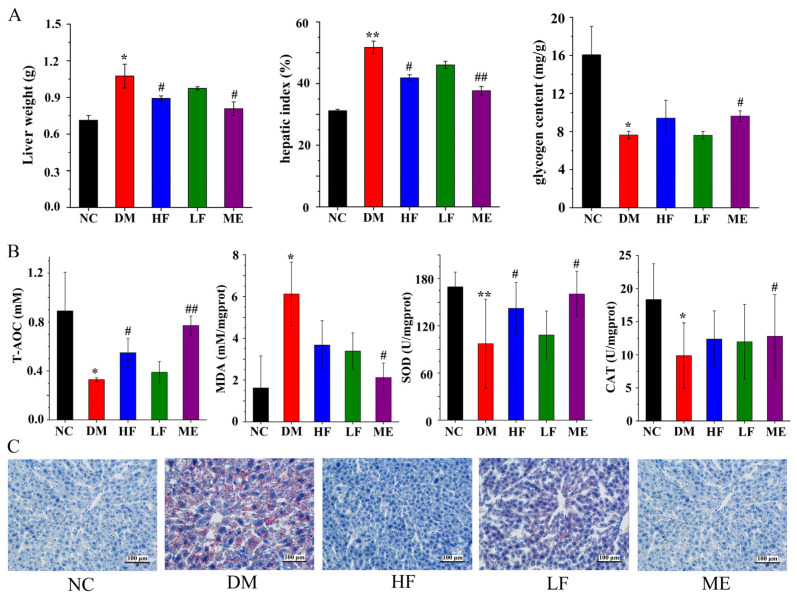
Protective effect of LJF on the liver: liver weight, hepatic index, and glycogen content (**A**); T-AOC, MDA, SOD, and CAT (**B**); histology analysis of liver by Oil-Red O dye staining (**C**). * *p* < 0.05 and ** *p* < 0.01 vs. NC group, and ^#^ *p* < 0.05 and ^##^ *p* < 0.01 vs. DM group.

The liver weight and hepatic index were distinctly increased in the DM group compared to the NC group, and hepatic glycogen was reduced (Figure 3A). LJF reduced liver weight and hepatic index in T2DM mice in a dose-dependent manner. Hepatic glycogen level was increased in the HF (*p* > 0.05) and ME groups compared to the DM group, and no difference was found between the LF and DM groups. As shown in Figure 3B, the contents of SOD, CAT, and T-AOC were distinctly reduced in the DM group compared to the NC group, and the MDA level was increased. High dosage of LJF reduced MDA level and increased T-AOC and SOD levels in T2DM mice, while low dosage of LJF reduced MDA level but had no effect on T-AOC, SOD, and CAT levels. Histology analysis showed that fat accumulation in the liver was distinctly increased in the DM group compared to the NC group, while LJF reduced fat accumulation in T2DM mice, especially high dosage.

### 3.4. LJF Regulated the Imbalance between Insulin Resistance and Insulin Sensitivity

**Figure 4:**
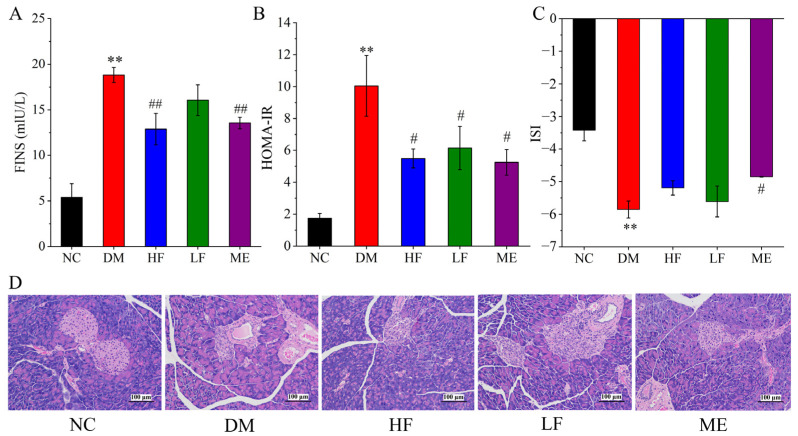
Effect of LJF on insulin activity: FINS (**A**), HOMA-IR (**B**), and ISI (**C**). Histology analysis of pancreas based on HE staining (**D**). ** *p* < 0.01 vs. NC group, and ^#^ *p* < 0.05 and ^##^ *p* < 0.01 vs. DM group.

As shown in Figure 4A, the level of FINS was distinctly increased in the DM group compared to the NC group, and LJF reduced FINS level in T2DM mice in a dose-dependent manner. A rise in HOMA-IR and a decline in ISI were found in the DM group, revealing the imbalance between insulin resistance and insulin sensitivity (Figure 4B,C). High dosage of LJF reduced HOMA-IR level and increased ISI level (*p* > 0.05) in T2DM mice, while low dosage of LJF reduced HOMA-IR level but had no effect on ISI level. It implies that the effect of LJF on the imbalance of insulin resistance and insulin sensitivity can be in a certain dose-dependent manner. Histology analysis showed that the structure of islet kept integrity in the NC group, and obvious pathological injuries were found in the pancreas islet of T2DM mice, including destruction of cell populations, inflammatory cells infiltration, and intracellular atrophy (Figure 4D). Low and high dosage of LJF reduced islet necrosis and β-cells damage, and the latter appeared to have better protective effect on the pancreas islet. These results suggest that LJF can protect the pancreas islet and regulate the imbalance of insulin resistance and insulin sensitivity to ameliorate the abnormality of insulin-glucose metabolism in T2DM mice.

### 3.5. LJF Increased the Production of SCFAs in T2DM Mice

**Figure 5:**
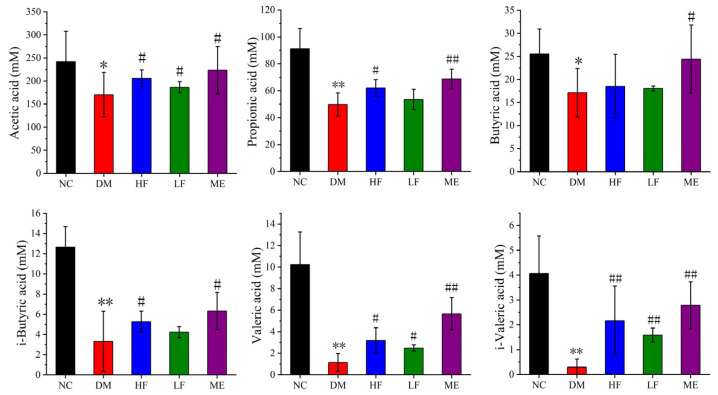
Effect of LJF on the SCFAs production, including acetic acid, propionic acid, butyric acid, i-butyric acid, valeric acid, and i-valeric acid. * *p* < 0.05 and ** *p* < 0.01 vs. NC group, and ^#^ *p* < 0.05 and ^##^ *p* < 0.01 vs. DM group.

The contents of SCFAs including acetic acid, butyric acid, propionic acid, i-butyric acid, i-valeric acid, and valeric acid were distinctly reduced in the DM group compared to the NC group (Figure 5). In contrast, high dosage of LJF increased the production of acetic acid, propionic acid, i-butyric acid, i-valeric acid, and valeric acid in T2DM mice, while low dosage of LJF increased the contents of acetic acid, valeric acid, and i-valeric acid. On the other hand, ME led to increases in acetic acid, butyric acid, propionic acid, i-butyric acid, i-valeric acid, and valeric acid in T2DM mice. High dosage of LJF had a similar effect with ME on the SCFAs, except butyric acid, implying their similarity on modulation of the gut microbiota.

### 3.6. LJF Modulated Dysbiosis of the Gut Microbiota in T2DM Mice

**Figure 6:**
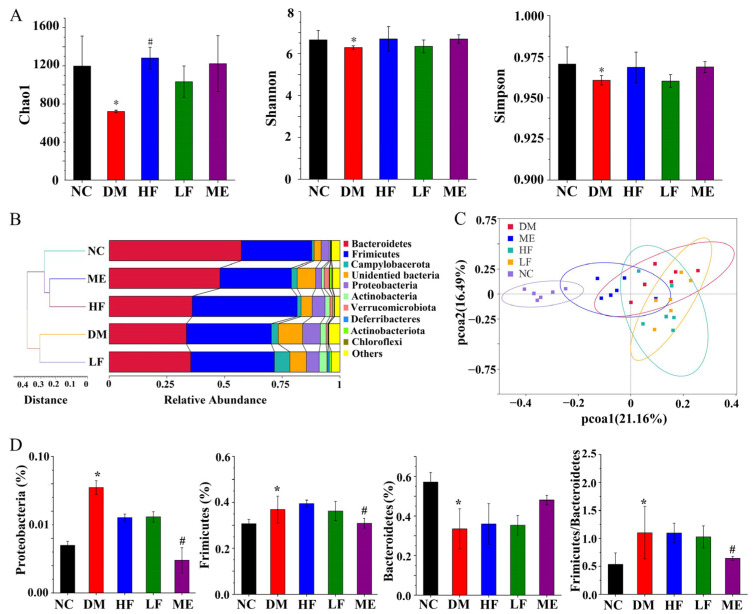
Effect of LJF on the gut microbiota: Chao1, Shannon, and Simpson indexes (**A**); UPGMA (**B**); PCoA (**C**); relative abundances of Proteobacteria, Firmicutes, Bacteroidetes, and the ratio of Firmicutes to Bacteroidetes at the phylum level (**D**). * *p* < 0.05 vs. NC group, and ^#^ *p* < 0.05 vs. DM group.

As shown in Figure 6A, the Chao1, Shannon, and Simpson indexes were reduced in the DM group compared to the NC group, and high dosage of LJF increased the Chao1 index, implying that high dosage of LJF can increase microbiota richness. UPGMA showed clustering between the LF and DM or HF and ME groups, and the HF and ME groups were closer to the NC group (Figure 6B). PCoA revealed an obvious separation between the NC and DM groups, and LJF and ME modulated the microbiota communities along the PCoA1 and PCoA2 axes (Figure 6C). At the phylum level, Firmicutes and Proteobacteria were increased in the DM group compared to the NC group, and Bacteroidetes was reduced, leading to an increased ratio of Firmicutes to Bacteroidetes. LJF reduced Proteobacteria level but had no effect on Firmicutes and Bacteroides in T2DM mice, and ME reduced Proteobacteria and Firmicutes levels and increased Bacteroidetes level, leading to a reduced ratio of Firmicutes to Bacteroides (Figure 6D). It implied that LJF and ME can modulate the gut microbiota composition with a certain specificity.

**Figure 7:**
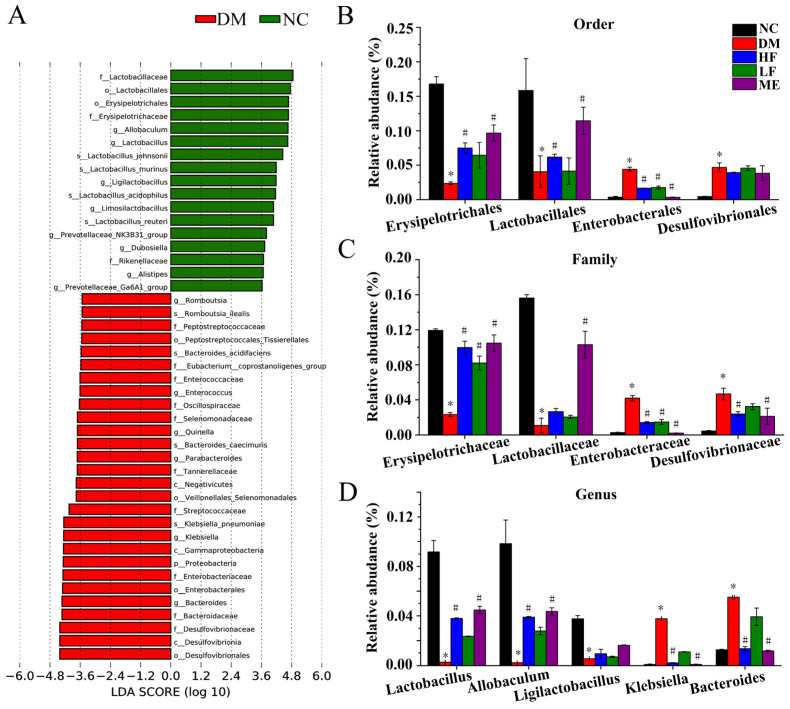
Analysis of key taxonomic taxa. LDA between the NC and DM groups (**A**). Comparative analysis of key bacteria at the order (**B**), family (**C**), and genus (**D**) levels. * *p* < 0.05 vs. NC group, and ^#^ *p* < 0.05 vs. DM group.

LDA showed that Lactobacillaceae, Lactobacillales, Erysipelotrichales, *Alistipes*, *Allobaculum*, Erysipelotrichaceae, *Lactobacillus*, *Ligilactobacillus*, and Rikenellaceae, and Prevotellaceae_NK3B31 and Ga6A1 groups were dominant in the NC group (Figure 7A), In contrast, *Romboutsia*, *Enterococcaceae*, *Enterococcus*, *Parabacteroides*, *Klebsiella*, *Bacteroides*, *Tannerellaceae*, *Desulfovibrionaceae*, *Streptococcaceae*, *Bacteroidaceae*, *Enterobacteriaceae*, *Enterobacterales,* and *Desulfovibrionales* were dominant in the DM group. Combined with pairwise comparative analysis (Figure S2), some key bacterial taxa were analyzed at the order, family, and genus levels. It showed that *Lactobacillales* and *Erysipelotrichales* were distinctly reduced in the DM group compared to the NC group, while *Enterobacterales* and *Desulfovibrionales* were increased. High dosage of LJF increased *Erysipelotrichales* and *Lactobacillales* and reduced Enterobacterales, and low dosage of LJF increased *Erysipelotrichales* and reduced Enterobacterales (Figure 7B). The same phenomenon was found in the dominant families within the above orders, *Erysipelotrichaceae*, *Lactobacillaceae*, *Enterobacteriaceae,* and *Desulfovibrionaeae* (Figure 7C). At the genus level, *Klebsiella* and *Bacteroides* were increased in the DM group compared to the NC group, and *Allobaculum*, *Lactobacillus,* and *Ligilactobacillus* were reduced (Figure 7D). LJF and ME increased *Lactobacillus* and *Allobaculum* and reduced *Klebsiella* and *Bacteroides*, and high dosage of LJF had better modulatory effect on the above genera compared to low dosage.

**Figure 8:**
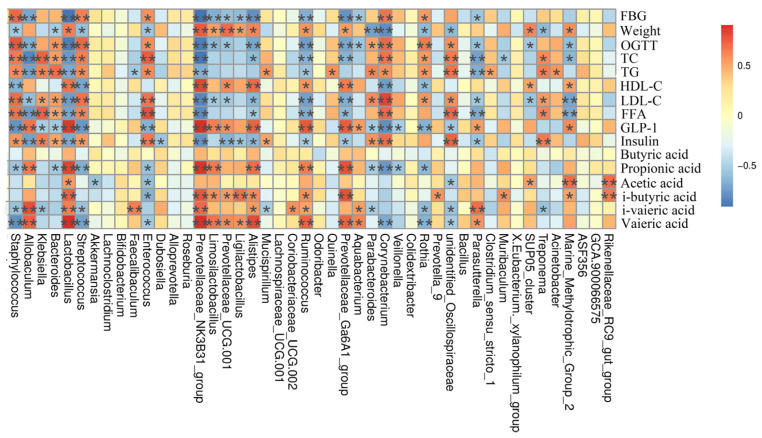
Spearman analysis between key bacterial genera and physiological indexes. * *p* < 0.05 and ** *p* < 0.05.

Spearman correlation analysis showed that *Bacteroides*, *Streptococcus*, *Staphylococcus*, *Rorthia, Enterococcus*, *Parabacteroides*, *Klebsiella,* and *Corynebacterium* were negatively correlated with SCFAs, GLP-1, HDL-C, and weight, and positively correlated with insulin, LDL-C, FFA, TC, OGTT, TG, and FBG (Figure 8). *Allobaculum*, *Alistipes*, *Lactobacillus*, Prevotellceae_UCG-001, NK3B31 and Ga6A1 groups, *Ligilactobacillus*, *Limosilactobacillus*, *Ruminococcus*, *Aquabaterium,* and *Parasutterella* were positively correlated with GLP-1, SCFAs, HDL-C, and weight, and had negative correlations with insulin, FFA, LDL-C, TG, TC, and FBG. *Faecalibaculum* was positively correlated with i-valeric acid and negatively correlated with TG. It implies that the effect of LJF can be associated with increases and reduction in some specific microbes, but the exact mechanism needs more in-depth study.

### 3.7. LJF Modulated the Microbiota Metabolites Profile in T2DM Mice

**Figure 9:**
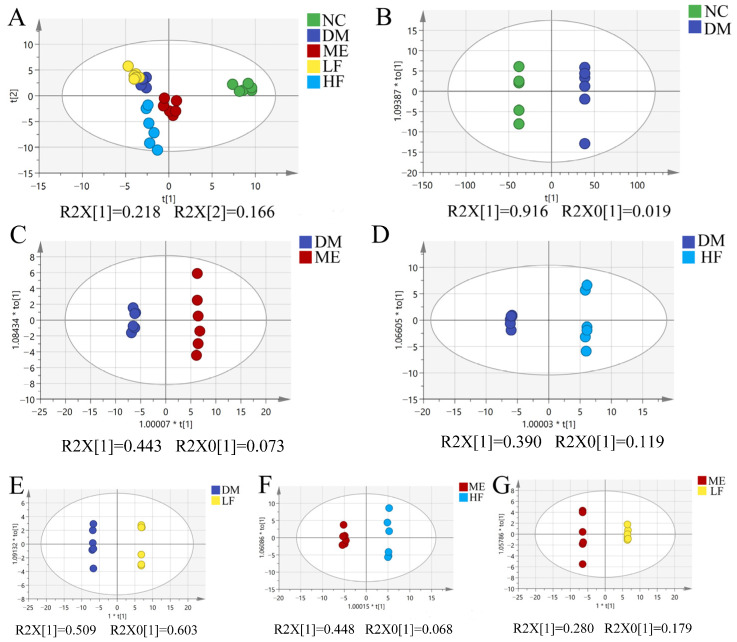
Untargeted metabonomics analysis of fecal samples by using AB Triple TOF 5600. PCA (**A**) and OPLS-DA between groups (**B**–**G**).

To understand the contribution of microbiota metabolites on the anti-diabetic effect of LJF, untargeted and targeted metabolomics were used to analyze the alterations of fecal metabolome induced by LJF. A total of 1135 metabolites were found in positive and negative modes by using the untargeted metabolomics method. PCA showed clustering and separation between the NC and DM groups, indicating that the metabolites profile was changed with microbiota dysbiosis (Figure 9A). However, no separations were found between the LF/HF/ME and DM groups. OPLS-DA was performed to further explore the relationship between the LF/HF/ME and DM groups (Figure 9B–G). Significant separation was found between the ME and DM groups, and the permutation test is shown in Figure S3 (R^2^ = 0.992; Q^2^ = −0.097). Obvious separations were also found between the LF/HF and DM groups, and R^2^ and Q^2^ were 0.987; −0.162 and 0.935; −0.059 respectively. It implied that the metabolites profile was changed with LJF-induced microbiota alterations. On the other hand, there were obvious separations between the LF/HF and ME groups, implying the difference of LJF and ME on modulation of the gut microbiota.

**Figure 10:**
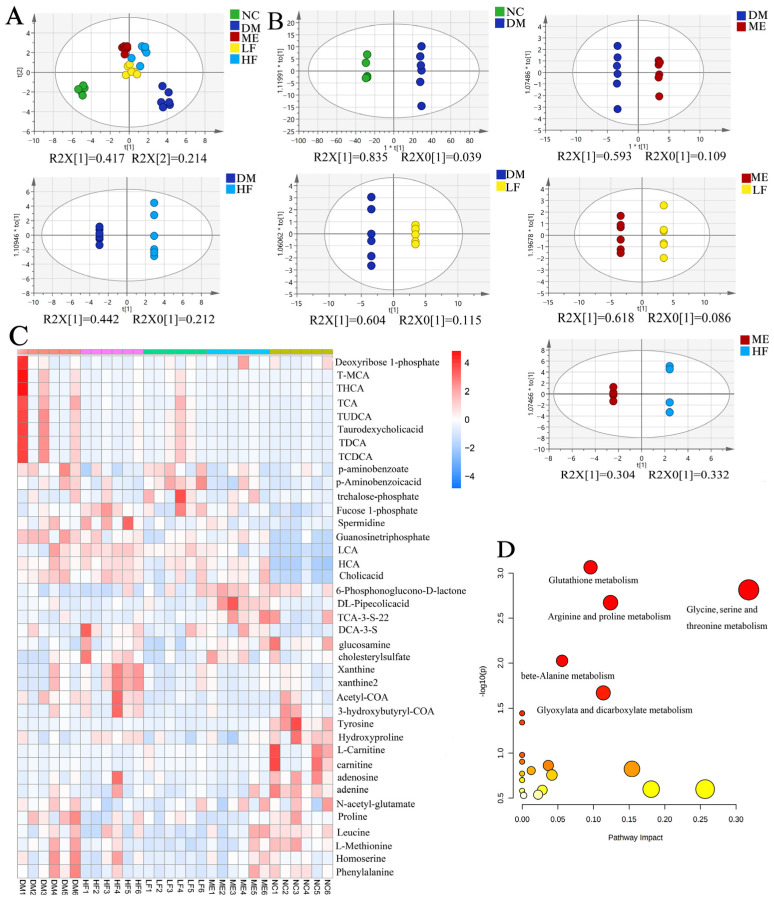
Targeted metabonomics analysis of fecal sample using AB 4000 QTRAP. PCA (**A**) and OPLS-DA (**B**). Heatmap of 39 metabolites with VIP > 1 (**C**). Analysis of metabolic pathways using Kyoto Encyclopedia of Genes and Genomes (**D**).

Then, targeted metabolomics analysis was used to identify altered metabolites, and a total of 104 metabolites were found in positive and negative modes. PCA showed a separation between the NC and DM groups, and mice fed with ME and LJF stayed away from the DM group (Figure 10A). OPLS-DA showed obvious separations between the DM and ME (R^2^ = 0.915; Q^2^ = −0.779)/HF (R^2^ = 1; Q^2^ = −0.613)/LF groups (R^2^ = 1; Q^2^ = −0.346), and obvious separations were also found between the ME and HF (R^2^ = 1; Q^2^ = −1.01)/LF (R^2^ = 1; Q^2^ = −0.379) groups (Figure 10B and Figure S4). The permutation tests verified that OPLS-DA models were reliable and had good predictability to screen the difference between groups. Thirty-nine metabolites were identified based on VIP > 1, and a heatmap was used to display their profiles in different groups (Figure 10C). The metabolites involved carbohydrates (organooxygen compounds and organic acids), proteins (amino acids, polyamines and enzymes), and fatty acids (esters and carnitine). LJF restored some metabolites toward to normal levels compared to the DM group, e.g., pipecolic acid, TUDCA, cholic acid, THCA, TCA, taurodeoxycholic acid, T-MCA, and homoserine. The major metabolic pathways involved glutathione, amino acids (arginine, threonine, proline, glycine, serine, and alanine), and glyoxylate and dicarboxylate metabolism pathways (Figure 10D).

### 3.8. Correlation Analysis between Bacterial Genera and Metabolites

**Figure 11:**
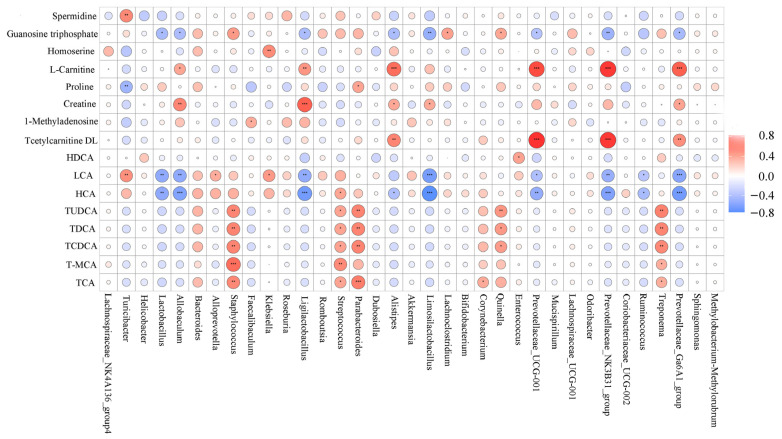
Spearman analysis between bacterial genera and microbial metabolites. * *p* < 0.05, ** *p* < 0.01 and *** *p* < 0.001.

LJF regulated gut microbiota dysbiosis in T2DM mice, thereby affecting the microbial metabolites profile. As shown in Figure 11, *Staphylococcus*, *Streptococcus*, *Bacteroides*, *Parabacteroides*, *Corynebacterium, Treponema,* and *Quinella* had positive correlations with TCA, TDCA, TUDCA, TCDCA, and T-MCA. LCA and HCA were positively correlated with *Staphylococcus, Turicibacter*, *Alloprevotella*, *Streptococcus, Klebsiella,* and *Bacteroides,* and negatively correlated with *Provotellaceae* subgroups, *Ligilactobacillus*, *Lactobacillus*, *Limosillactobacillus*, *Alistipes, Allobaculum,* and *Ruminococcus*. Tcetylarnitine DL was positively correlated with Provotellaceae subgroups and *Alistipes*, and creatine and L-carnitine were positively correlated with *Limosillactobacillus, Ligilactobacillus*, *Allobaculum,* Provotellaceae subgroups, and *Alistipes.* In addition, Guanosine triphosphate was negatively correlated with Provotellaceae subgroups, *Lactobacillus*, *Limosillactobacillus, Allobaculum*, *Alistipes,* and *Ligilactobacillus* and positively correlated with *Staphylococcus, Quinella,* and *Lachnoclostridium*. Spermidine was positively correlated with *Turicibacter*. It showed close relationships between microbial metabolites and gut microbes, but the underlying mechanisms of microbial metabolites on the host need more study.

## 4. Discussion

Diabetes has been one of the biggest threats to human health, the incidence of which is increasing all over the world. Current drugs effectively reduce the mortality and morbidity of diabetes, but their adverse effects on human health is becoming an urgent issue [5]. For people with diabetes, the most challenging part is the control of their dietary pattern, which plays an important role in the prevention and management of diabetes [20]. Accumulating studies indicate that polysaccharides can benefit diabetes and related complications, indicating that it can be used for the prevention and treatment of diabetes [21]. 

Weight loss is the typical symptom of T2DM, which can be attributed to the loss of appetite and tissue protein. LJF reduced water intake and increased weight gain in T2DM mice, which was consistent with pumpkin polysaccharides [22]. Lower glucose tolerance, higher FBG level, insulin resistance, and dyslipidemia are the basic pathologic characteristics of T2DM [23]. LJF reduced blood glucose levels in T2DM mice, which could be attributed to the improved balance between glucose absorption and utilization [24]. LJF not only regulated TG, TC, HDL-C, and LDL-C contents, but also reduced ALT, AST, and FFA levels and elevated GLP-1 level in T2DM mice. GLP-1 plays a critical role in insulin secretion and food intake due to its powerful insulinotropic effect [24]. A rise in FFA has a lipotoxic effect on the pancreas, which contributes to insulin resistance in the liver and the damage of islet cells [25]. Serum insulin was higher in T2DM mice, which can be explained by recognizing that serum insulin was increased with insulin resistance to reduce blood glucose level [26]. LJF reduced HOMA-IR index and increased ISI index in T2DM mice, indicating that LJF improved the balance between insulin resistance and insulin sensitivity, which was same with *Arimillariella tabescens* polysaccharides [16]. On the other hand, LJF reduced islet central necrosis and β cells damage, thereby improving insulin-glucose metabolism.

Diabetes is associated with excessive production of reactive oxygen species (ROS), leading to oxidative damage in the liver and defects in insulin action and secretion [27]. The metabolic increase due to mitochondrial glucose oxidation affects glucose and lipid peroxidation and LDL-C level. Excess ROS can react rapidly with polyunsaturated fatty acids to form lipid peroxides and exert cytotoxic effect on phospholipids, causing MDA formation. MDA is a key indicator reflecting the degree of oxidation in the body. ROS that generated in the tissues can be scavenged by enzymatic antioxidants (e.g., CAT and SOD) and non-enzymatic antioxidants. The superoxide radical is converted to H_2_O_2_ by SOD, and H_2_O_2_ is detoxified to H_2_O and O_2_ by CAT. LJF reduced MDA level and increased T-AOC and SOD levels, which was consistent with pumpkin polysaccharides protecting against oxidative damage in the liver via increasing SOD and GSH levels and reducing MDA and ROS levels. 

Gut microbiota plays a key role in the post-natal maturation of the immune system, food digestion, regulation of endocrine functions, and elimination of toxins [28]. Dysbiosis of gut microbiota is associated with some diseases, e.g., obesity and T2DM [29]. However, some reports varied regarding the association of taxonomic groups with diseases. A comparative study of the gut microbiota in patients with T2DM indicated that low diversity of gut microbiota was closely related to a higher prevalence of insulin resistance [30,31]. LJF increased microbiota diversity and richness and reduced Proteobacteria, which was similar to tea polysaccharides increasing microbiota diversity and modulating the ratio of Firmicutes to Bacteroidetes [32]. The ratio of Firmicutes to Bacteroidetes is recognized as a marker of some diseases, but there was no consistent association between this ratio and T2DM [33]. Moreover, increased Proteobacteria is recognized as a marker for an unstable microbiota community and a potential diagnostic criterion for some diseases [34]. It implied that the anti-diabetic effect of LJF can be associated with structural alteration of gut microbiota and reduction in Proteobacteria.

Human studies revealed the association of taxonomic groups and some phenotypes of T2DM, in which a few common results were found [33]. *Akkermansia*, *Faecalibacterium*, *Bacteroides*, *Roseburia,* and *Bifidobacterium* were negatively correlated with T2DM, and *Fusobacterium*, *Blautia,* and *Ruminococcus* had positive associations with T2DM. LJF increased the levels of *Lactobacillus* and *Allobaculum* and reduced *Klebsiella* and *Bacteroides* levels in T2DM mice. It was consistent with mulberry fruit polysaccharides increasing the levels of *Lactobacillus* and *Allobaculum* in T2DM mice [35]. Accumulating studies have shown that *Lactobacillus* alleviated liver damage and insulin resistance in T2DM mice due to its activities and cellular components [36,37]. Additionally, diabetic mice treated with ME had a higher level of *Akkermansia*, which was associated with enhanced glucose tolerance and reduced inflammation [38]. However, this phenomenon was not found in T2DM mice fed with LJF. To date, it is hard to define clearly a causality between specific bacteria and T2DM, but it is no doubt that the gut microbiota plays an important role in the occurrence and attenuation of T2DM.

SCFAs are generated by the microbial fermentation of polysaccharides, which play a key role in host health, e.g., intestinal functions and immune response [39]. A recent study indicated that the beneficial effect of SCFAs on glucose-lipid metabolism can be attributed to its regulation of the functions of metabolically active organs [40]. SCFAs can affect energy metabolism via a series of signaling pathways mediated by G-protein-coupled receptors and intestinal peptides from L cells [32]. LJF increased SCFA production in the colon, which was consistent with tea polysaccharides reducing serum glucose and lipids in T2DM mice and accompanied by modulation of gut microbiota and increases in SCFAs [32]. Spearman analysis suggested that *Allobaculum*, *Lactobacillus*, *Ruminococcus*, *Prevotellaceae,* and *Rikenellaceae* subgroups and *Alistipes* can play key roles in T2DM via SCFAs-related pathways. *Alistipes* is a relatively new genus and highly relevant in microbiota dysbiosis and diseases, and SCFAs are its important metabolites [41]. *Allobaculum* and *Lactobacillus* can play important roles in mitigating high fat diet-induced obesity, which can be associated with increased SCFAs content in the colon [42].

Most microbiota metabolites can be used as signaling molecules to participate in life activities, and their changes are considered as key risks for health. LJF led to a separation of the microbial metabolite profile from T2DM, and 39 metabolites were changed, mainly involving amino acids, glutathione, and glyoxylate and dicarboxylate metabolism pathways. Recent studies showed that specific amino acids were associated with the risk of developing T2DM, e.g., serine, aromatic, and branched-chain amino acids [43,44]. Specific amino acids can stimulate β-cells electrical activity that is essential for insulin secretion in the presence of glucose, e.g., leucine, alanine, isoleucine, and arginine [45]. Clinical study showed that arginine reduced weight gain, decreased blood pressure, normalized endothelial dysfunction, and ameliorated T2DM [46]. Glutathione is the most abundant intracellular antioxidant, involving β-cell dysfunction and some T2DM complications [47]. Glutathione is a tripeptide synthesized from glutamate, cysteine, and glycine, and its deficiency can be restored by glutathione supplementation [48] or its precursor amino acids [49]. Glyoxylate and dicarboxylate metabolism is linked to obesity, atherosclerosis, and diabetes, which can be attributed to the modulation of SCFAs, amino acids, and glutamate metabolism [50]. Previous study showed that *Lycium barbarum* polysaccharides regulated glucose and liver metabolism in T2DM mice, and glyoxylate and dicarboxylate metabolism was one of the major altered metabolic pathways [51]. 

## 5. Conclusions

This study indicates that LJF can improve the symptoms of T2DM, e.g., body weight, glucose and lipid metabolism, oxidative stress, and pancreatic islet integrity, and a dose-response effect was found among them. The underlying mechanisms can be associated with modulation of the gut microbiota, e.g., increases in *Lactobacillus* and *Allobaculum*. In addition, microbial metabolites play an irreplaceable role in the remission of T2DM, mainly involving amino acids, glutathione, and glyoxylate and dicarboxylate metabolism pathways. Results indicated that LJF can be developed as a prebiotic agent for the prevention and treatment of T2DM. However, some key issues need to be further explored before its application, e.g., key bacteria and regulatory mechanisms of LJF on the gut microbiota.

## Data Availability

Data is contained within the article or supplementary material.

## Supplementary Materials

The following supporting information can be downloaded at: https://www.mdpi.com/article/10.3390/foods12010033/s1, Figure S1: Monosaccharide composition of LJF and infrared spectrogram; Figure S2: Pairwise comparative analysis between groups using LDA; Figure S3: Permutation tests of OPLS-DA in the untargeted method; Figure S4: Permutation tests of OPLS-DA in the targeted method; Table S1: Basic characteristics of LJF.

## Abbreviation

LJF	Laminaria japonica fucoidan
HFD	High fat diet
STZ	Streptozocin
T2DM	Type 2 diabetes mellitus
FBG	Fasting blood glucose
OGTT	Oral glucose tolerance test
TC	Total cholesterol
TG	Triglyceride
HDL-C	High density lipoprotein cholesterol
LDL-C	Low density lipoprotein cholesterol
ALT	Alanine transaminase
AST	Aspartate aminotransferase
FINS	Fasting serum insulin
HOMA	Homeostasis model assessment
IR	Insulin resistance
ISI	Insulin sensitivity index
FFA	Free fatty acids
GLP-1	Glucagon-like peptide 1
SOD	Superoxide dismutase
MDA	Malondialdehyde
T-AOC	Total antioxidant capacity
CAT	Catalase
SCFAS	Short chain fatty acids
TCA	Tricarboxylic acid
TDCA	Taurodeoxycholic acid
TUDCA	Tauroursodeoxycholic acid
TCDCA	Taurochenodeoxycholic acid
T-MCA	Tauro-muricholic acid
LCA	Lymphocyte common antigen
HCA	Hydroxycitric acid
