# Correction: Zhang et al. Fucoidan from *Laminaria japonica* Ameliorates Type 2 Diabetes Mellitus in Association with Modulation of Gut Microbiota and Metabolites in Streptozocin-Treated Mice. *Foods* 2023, *12*, 33

**DOI:** 10.3390/foods12224132

**Published:** 2023-11-15

**Authors:** Chenxi Zhang, Jinhui Jia, Panpan Zhang, Weiyun Zheng, Xiaoming Guo, Chunqing Ai, Shuang Song

**Affiliations:** 1 School of Food Science and Technology, National Engineering Research Center of Seafood, Dalian Polytechnic University, Dalian 116034, China; 2 Shenzhen Key Laboratory of Food Nutrition and Health, Institute for Advanced Study, Shenzhen University, Shenzhen 518060, China; 3 National & Local Joint Engineering Laboratory for Marine Bioactive Polysaccharide Development and Application, Dalian Polytechnic University, Dalian 116034, China

## Error in Figure

In the original publication, there was a mistake in Figure 3 as published [1]. In Figure 3, a tissue section (ME) stained with Oil red O was reused in the process of picture organization. The corrected Figure 3 appears below. The authors state that the scientific conclusions are unaffected. This correction was approved by the Academic Editor. The original publication has also been updated.

## Figures and Tables

**Figure 3:**
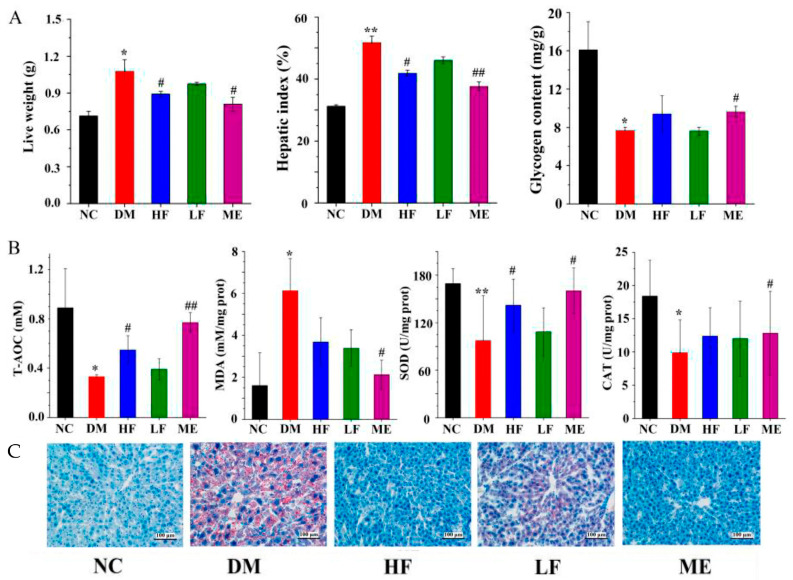
Protective effect of LJF on the liver: liver weight, hepatic index, and glycogen content (**A**); T-AOC, MDA, SOD, and CAT (**B**); histology analysis of liver by Oil-Red O dye staining (**C**). * *p* < 0.05 and ** *p* < 0.01 vs. NC group, and *# p* < 0.05 and *## p* < 0.01 vs. DM group.
