# Supplementary material for: Fucoidan from Laminaria japonica Ameliorates Type 2 Diabetes Mellitus in Association with Modulation of Gut Microbiota and Metabolites in Streptozocin-Treated Mice
Source: Foods. 2022 Dec 22;12(1):33. doi: 10.3390/foods12010033 (PMC9818518; doi:10.3390/foods12010033)
Supplement: Supplementary file 1 [file foods-12-00033-s001.zip › foods-2068864-supplementary.pdf]

Table S1

| Protein (%) | Uronic acid (%) | Sulfate group (%) | Total sugar (%) | Molecular weight |
|---|---|---|---|---|
| 3.70 ± 0.5 | 17.90 ± 0.7 | 19.53 ± 0.4 | 68.8 ± 0.4 | 89 kDa |

Protein was quantified through the Bradford method with BSA as standard. Total sugar and uronic acid content were determined by using the phenol-sulfuric acid method and meta-hydroxydiphenyl-sulfuric acid method respectively (doi:10.1016/j.carbpol.2010.12.033). The level of sulfate group was measured through the barium chloride-gelatin method using $K_2SO_4$ as standard (doi: 10.1042/bj0840106).

2.0 mg of LJF dissolved in 1.0 mL of 100 mM ammonium acetate solution. After vortex oscillation, the solution passed 0.22 μM hole membrane filter filtration (JINTENG, Tianjin, China). Relative molecular weight of LJF was determined by high-performance gel permeation chromatography (HPGPC) using a Waters Alliance 2695 HPLC (Milford, MA, USA) system equipped with a Waters Alliance 2414 refractive index detector and analytical column of TSK-G2500PWXL (7.8 × 300 mm, Tosoh Corporation, Tokyo, Japan) at 30◦C. The mobile phase was 100 mM ammonium acetate solution (pH 6.0) with a flow rate of 0.4 mL/min, and the injection volume was 10 μL. A standard curve was plotted with the value of average relative molecular weight on the retention time using Dextran (1, 5, 25, 50 and 150 kDa) as standard (doi:10.1016/j.foodres.2022.110980).

Figure S1

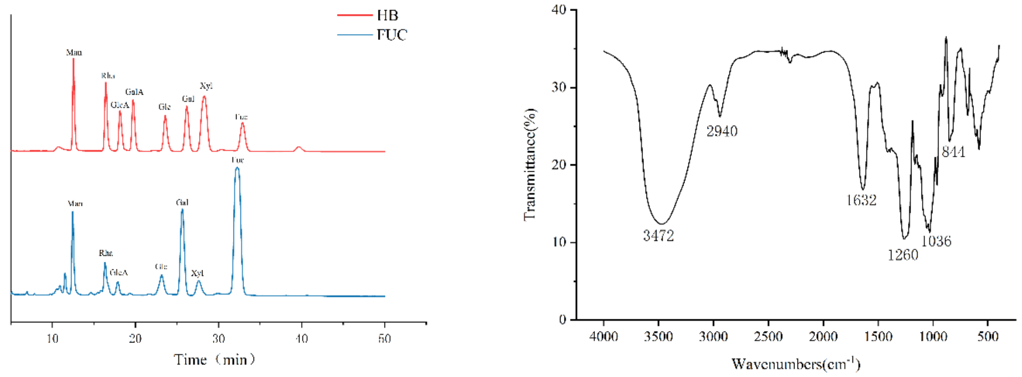


The monosaccharides composition of LJF was determined using high-performance liquid chromatography (HPLC) analysis of monosaccharide derivatives with 1-phenyl-3-methyl-5-pyrazolone in sample hydrolysates as previous study ( doi:10.1016/j.carbpol.2010.05.029).

The samples were ground with dried potassium bromide powder and pressed into 1 mm pellets for FT-IR spectrophotometer (Perkin Elmer, Waltham, MA, USA) measurements in the range of 400 ~ 4000cm$^{-1}$ (doi:10.1016/j.foodres.2022.110980). The FT-IR spectrum of LJF was shown in Figure. A1 right. The absorption peaks at 3472 and 2940 cm$^{-1}$ were assigned to O-H and C-H stretching vibrations respectively. 1632cm$^{-1}$ were contributed to the carboxyl group. The absorption peak at 1260cm$^{-1}$ was considered as stretching vibration of S=O of sulfate group. The peaks between 1036 and 844cm$^{-1}$ belonged to stretching vibrations of the C-O bond.

Figure S2

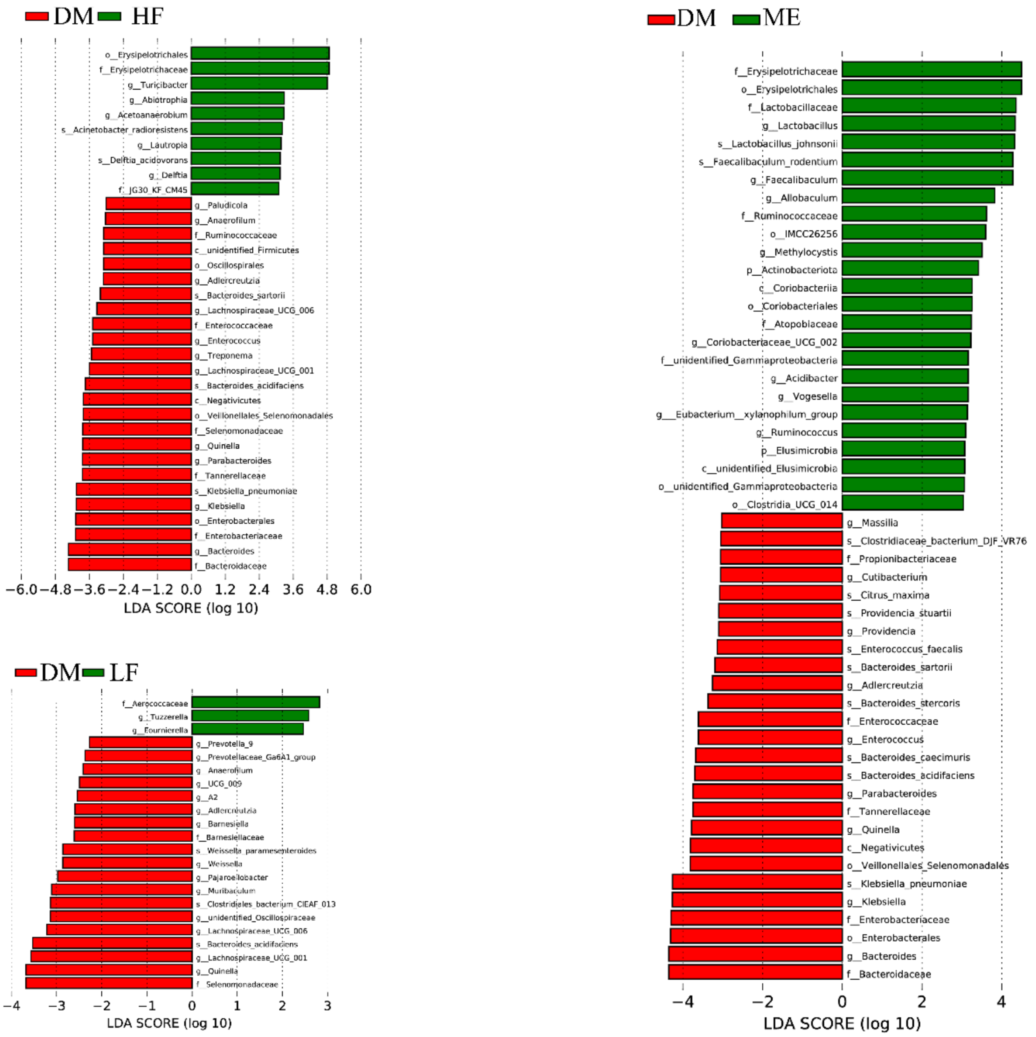
DM
HF
o__Erysipelotrichales
f__Erysipelotrichaceae
g__Turicibacter
g__Abiotrophia
g__Acetoanaerobium
s__Acinetobacter_radioresistens
g__Lautropia
s__Delftia_acidovorans
g__Delftia
f__JG30_KF_CM45
g__Paludicola
g__Anaerofilum
f__Ruminococcaceae
c__unidentified_Firmicutes
o__Oscillospirales
g__Adlercreutzia
s__Bacteroides_sartorii
g__Lachnospiraceae_UCG_006
f__Enterococcaceae
g__Enterococcus
g__Treponema
g__Lachnospiraceae_UCG_001
s__Bacteroides_acidifaciens
c__Negativicutes
o__Veillonellales_Selenomonadales
f__Selenomonadaceae
g__Quinella
g__Parabacteroides
f__Tannerellaceae
s__Klebsiella_pneumoniae
g__Klebsiella
o__Enterobacterales
f__Enterobacteriaceae
g__Bacteroides
f__Bacteroidaceae
−6.0 −4.8 −3.6 −2.4 −1.2 0.0 1.2 2.4 3.6 4.8 6.0
LDA SCORE (log 10)
DM
LF
f__Aerococcaceae
g__Tuzzerella
g__Fournierella
g__Prevotella_9
g__Prevotellaceae_Ga6A1_group
g__Anaerofilum
g__UCG_009
g__A2
g__Adlercreutzia
g__Barnesiella
f__Barnesiellaceae
s__Weissella_paramesenteroides
g__Weissella
g__Pajaroellobacter
g__Muribaculum
s__Clostridiales_bacterium_CIEAF_013
g__unidentified_Oscillospiraceae
g__Lachnospiraceae_UCG_006
s__Bacteroides_acidifaciens
g__Lachnospiraceae_UCG_001
g__Quinella
f__Selenomonadaceae
−4 −3 −2 −1 0 1 2 3
LDA SCORE (log 10)
DM
ME
f__Erysipelotrichaceae
o__Erysipelotrichales
f__Lactobacillaceae
g__Lactobacillus
s__Lactobacillus_johnsonii
s__Faecalibaculum_rodentium
g__Faecalibaculum
g__Allobaculum
f__Ruminococcaceae
o__IMCC26256
g__Methylocystis
p__Actinobacteriota
c__Coriobacteriia
o__Coriobacteriales
f__Atopobiaceae
g__Coriobacteriaceae_UCG_002
f__unidentified_Gammaproteobacteria
g__Acidibacter
g__Vogesella
g___Eubacterium__xylanophilum_group
g__Ruminococcus
p__Elusimicrobia
c__unidentified_Elusimicrobia
o__unidentified_Gammaproteobacteria
o__Clostridia_UCG_014
g__Massilia
s__Clostridiaceae_bacterium_DJF_VR76
f__Propionibacteriaceae
g__Cutibacterium
s__Citrus_maxima
s__Providencia_stuartii
g__Providencia
s__Enterococcus_faecalis
s__Bacteroides_sartorii
g__Adlercreutzia
s__Bacteroides_stercoris
f__Enterococcaceae
g__Enterococcus
s__Bacteroides_caecimuris
s__Bacteroides_acidifaciens
g__Parabacteroides
f__Tannerellaceae
g__Quinella
c__Negativicutes
o__Veillonellales_Selenomonadales
s__Klebsiella_pneumoniae
g__Klebsiella
f__Enterobacteriaceae
o__Enterobacterales
g__Bacteroides
f__Bacteroidaceae
−4 −2 0 2 4
LDA SCORE (log 10)

Figure S3

A

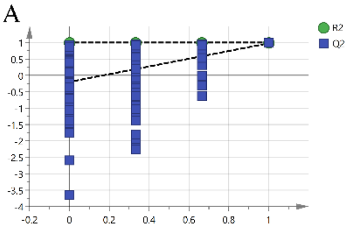


NC-DM R2=1;Q2=-0.23

B

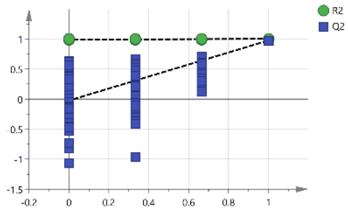


DM-ME R2=0.992;Q2=-0.097

C

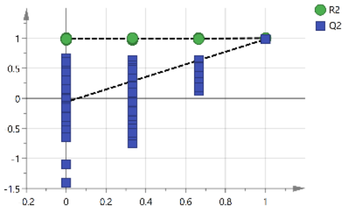


DM-LF R2=0.987;Q2=-0.162

D

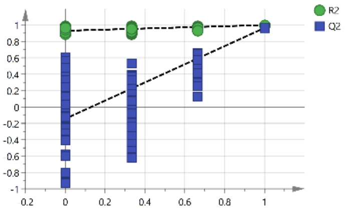


DM-HF R2=0.935;Q2=-0.059

E

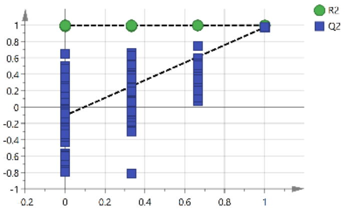


ME-HF R2=0.992;Q2=-0.126

F

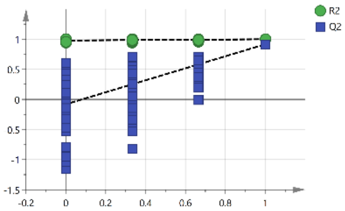


ME-LF R2=0.978;Q2=-0.079

Figure S4

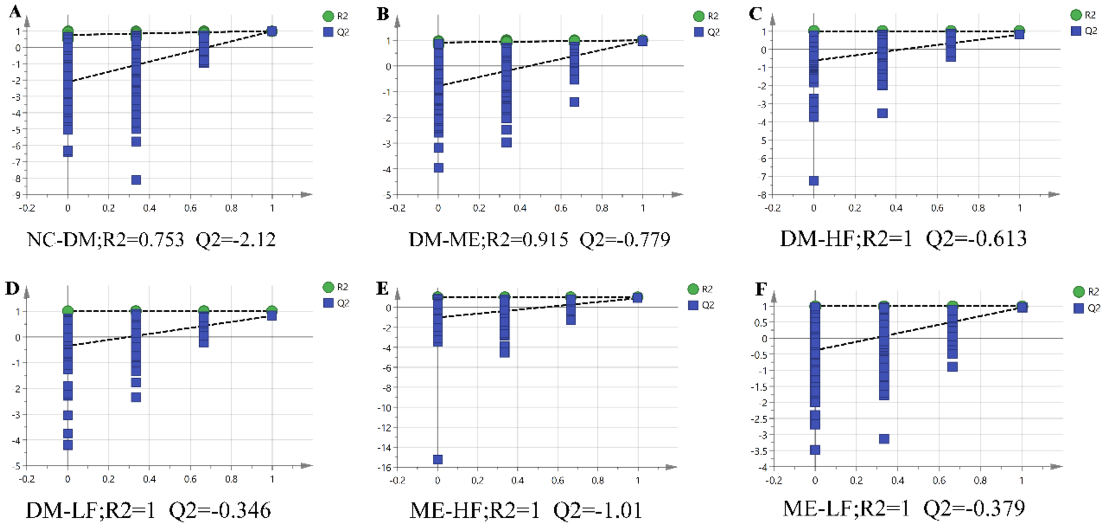
A
R2
Q2
NC-DM;R2=0.753  Q2=-2.12
B
DM-ME;R2=0.915  Q2=-0.779
C
DM-HF;R2=1  Q2=-0.613
D
DM-LF;R2=1  Q2=-0.346
E
ME-HF;R2=1  Q2=-1.01
F
ME-LF;R2=1  Q2=-0.379
